# The self-reported health of U.S. flight attendants compared to the general population

**DOI:** 10.1186/1476-069X-13-13

**Published:** 2014-03-10

**Authors:** Eileen McNeely, Sara Gale, Ira Tager, Laurel Kincl, Julie Bradley, Brent Coull, Steve Hecker

**Affiliations:** 1 Department of Environmental Health, Harvard School of Public Health, Building 1, Room 1401, 655 Huntington Avenue, Boston, MA, USA; 2 Division of Epidemiology, School of Public Health, University of California, Berkeley, 50 University Hall, Berkeley, CA 94720-7360, USA; 3 Oregon State University, College of Public Health and Human Sciences, 116 Milam Hall, Corvallis, OR 97331-6102, USA; 4 University of Oregon, Labor Education and Research Center, 1675 Agate St, Eugene, OR 97403, USA

**Keywords:** Flight attendant health, Occupational diseases in airliner cabin crew, Flight attendant jobs, Chronic bronchitis, Depression, Fatigue, Sleep disorders, Hearing loss, Heart disease, Cancer, Second-hand tobacco smoke exposure

## Abstract

**Background:**

Few studies have examined the broad health effects of occupational exposures in flight attendants apart from disease-specific morbidity and mortality studies. We describe the health status of flight attendants and compare it to the U.S. population. In addition, we explore whether the prevalence of major health conditions in flight attendants is associated with length of exposure to the aircraft environment using job tenure as a proxy.

**Methods:**

We surveyed flight attendants from two domestic U.S. airlines in 2007 and compared the prevalence of their health conditions to contemporaneous cohorts in the National Health and Nutrition Survey (NHANES), 2005-2006 and 2007-2008. We weighted the prevalence of flight attendant conditions to match the age distribution in the NHANES and compared the two populations stratified by gender using the Standardized Prevalence Ratio (SPR). For leading health conditions in flight attendants, we analyzed the association between job tenure and health outcomes in logistic regression models.

**Results:**

Compared to the NHANES population (n =5,713), flight attendants (n = 4,011) had about a 3-fold increase in the age-adjusted prevalence of chronic bronchitis despite considerably lower levels of smoking. In addition, the prevalence of cardiac disease in female flight attendants was 3.5 times greater than the general population while their prevalence of hypertension and being overweight was significantly lower. Flight attendants reported 2 to 5.7 times more sleep disorders, depression, and fatigue, than the general population. Female flight attendants reported 34% more reproductive cancers. Health conditions that increased with longer job tenure as a flight attendant were chronic bronchitis, heart disease in females, skin cancer, hearing loss, depression and anxiety, even after adjusting for age, gender, body mass index (BMI), education, and smoking.

**Conclusions:**

This study found higher rates of specific diseases in flight attendants than the general population. Longer tenure appears to explain some of the higher disease prevalence. Conclusions are limited by the cross-sectional design and recall bias. Further study is needed to determine the source of risk and to elucidate specific exposure-disease relationships over time.

## Background

The health of U.S. flight attendants, a workforce of 84,960 in 2012 [1], has not been well characterized. Change in the airline industry over the past few decades has further complicated the understanding of occupational health risks. Flight attendants are older and more diverse than in the past, and the job has changed dramatically [2,3]. The work now includes longer flight times with quicker turnaround times between flights, circumpolar navigational routes, increased passenger loads in new jumbo-sized planes and increased occupancy aboard all flights, and new security procedures. These conditions may strain customer relations [4,5], add to circadian rhythm disruption [6-8], and intensify known occupational exposures such as the physical demands of work in restricted cabin quarters, cosmic radiation, cabin air contaminants, low pressure and humidity, noise, vibration, and gravitational forces [9,10].

Investigation of the flight attendants’ occupational exposures is limited. Studies about the potential effects of cosmic radiation and past exposure to tobacco smoke in the aircraft cabin provide some insights. The accumulated evidence weighs arguably towards flight attendants being at higher risk of certain cancers, such as breast and skin cancers, although not all studies support this finding [11-20]. In addition, flight attendants exposed to tobacco smoke in the cabin were found to have higher rates of respiratory disease, although only a few studies have followed respiratory outcomes much beyond the early years of the smoking ban, now over a decade old [21-26]. There are no recent studies that profile the scope and severity of health conditions of flight attendants beyond a few survey studies limited by either non-random selection or low participation [9,21,27-30].

Importantly, a thorough understanding of occupational risk is needed since currently employed flight attendants have been in their jobs longer than any previous airline workforce. Longer tenure followed successful challenges to discriminatory terminations in the 1970s and hiring freezes brought about by deregulation and consolidation in the industry [2,3]. Longer tenure very likely means longer exposure to potential occupational hazards. When exposure data are not available for study subjects in an occupational cohort, employment tenure or job duration can serve as a surrogate for cumulative exposure to occupational hazards with the assumption of a uniform exposure intensity, which does not change over time or across study participants [31]. Several aviation studies use tenure as a proxy for exposure [15], and tenure often correlates with radiation exposure [32,33].

To address the gaps in information about flight attendant health, we conducted a large, cross-sectional survey of flight attendant health with three main aims: (1) to profile prevalent health conditions in flight attendants in a random sample of the population using a survey; (2) to compare the health experience of flight attendants to a nationally representative sample from the National Health and Nutrition Examination Survey (NHANES) using standardized prevalence ratios for health conditions that were included in both surveys; and (3) to investigate the relationship between the most prevalent health conditions in flight attendants and the duration of exposure to aircraft cabin environment using employment tenure as a proxy for exposure time.

## Methods

With the assistance of the Association of Flight Attendants-CWA, AFL-CIO union, we surveyed flight attendants employed by two domestic carriers and based at any one of five major city hubs in the west, central, and mid-eastern United States. In 2007, we mailed surveys to a random selection of the flight attendant population and supplemented the mailing by distributing and collecting surveys at the airport hubs before flight departures or after flight arrivals. To each flight attendant selected at random from union membership lists, we mailed two surveys and two reminder postcards. Also, researchers visited the five target airport hubs across the country and met some of those flight attendants already contacted via mail and reminded them to mail back or return the surveys directly to the research team in the field. In addition, new participants outside of the survey mailing lists were recruited to participate at the time of our on-site reminder/recruitment campaign. In the few cases of duplicate surveys, such as when flight attendants completed a mailed survey and another survey from the on-site airport distribution, we accepted only the mailed survey returned by the flight attendant. In the final study sample, participants selected at random outnumbered the participants we added at the airports 2:1. The Harvard School of Public Health Institutional Review Board approved all protocols for human subjects.

The survey was constructed using standardized questions from other surveys (Job Content Questionnaire [34], Centers for Disease Control – National Center for Health Statistics (CDC-NCHS), National Health and Nutrition Examination Survey (NHANES) (CDC-NCHS 2005-2008)) and feedback generated from focus groups and a pilot study sample.

To understand how the health of flight attendants compares to the general population, we used data from the National Health and Nutrition Examination Surveys (NHANES) for the survey years 2005-2006 and 2007-2008. NHANES is a program from the National Center for Health Statistics (NCHS) within the Centers for Disease Control and Prevention (CDC). NCHS designed the survey to obtain demographic, health, dietary, and laboratory data from a representative sample of approximately 5,000 US residents every year. After an extensive side-by-side review of both flight attendant and NHANES surveys, we selected common questions in the NHANES using data from the demographic, blood pressure, current health status, medical conditions, sleep disorder, smoking, and household smoking sections of the NHANES questionnaire (Additional file 1: Table S1). Most of the questions between the FA survey and NHANES questionnaire aligned to binary answer choices for prevalence (yes/no), but the fatigue and depression variables contained slightly different time interval answer choices. To estimate the prevalence for fatigue and depression, we used a conservative approach and coded symptoms over the past week that occurred “every day (7 days)” in the flight attendants as a “yes” for fatigue or depression, and only the category of “nearly every day” over the past 2 weeks in NHANES as a “yes” for these same conditions. We weighted the NHANES data by their four-year sample weights, primary sampling units, and strata according to the NHANES analytic guidelines [35]. To match the characteristics of the flight attendants, we limited the NHANES respondents to participants 18 years old and over, a family income to poverty ratio of 1 or greater, a high school/GED education or greater, and current employment.

To compare the two populations, we chose the Standardized Prevalence Ratio (SPR), which is structured the same as a Standardized Mortality Ratio (SMR), an indirect method of standardization in epidemiology [36]. The SPR is weighted by age (18-39, 40-59, and ≥ 60 years) and analyzed separately for males and females. The SPR is a comparison of the observed to the expected prevalence of disease. To calculate the SPR, we use the prevalence of a health condition in the flight attendant population as the observed total cases, and the expected total cases are calculated with the prevalence from the NHANES survey applied to the flight attendant population.

To test the relation between job exposures and the prevalence of disease in flight attendants, we used logistic regression. To predict the odds of disease with each five-year increment in job tenure (years on the job), we stratified the analysis by gender and adjusted for risk factors such as age, education, body mass index and current smoking. Analysis was completed using STATA statistical software, version 10 (StataCorp, College Station, Texas).

## Results

We collected 4,011 flight attendant surveys, which included 2,613 surveys (48% response rate) from flight attendants who were randomly selected to receive a survey in the mail. In addition, we collected another 1,398 surveys at the airports from a convenience sample of flight attendants employed by the same airlines. In all, we collected surveys from the equivalent of one-third of all flight attendants on the airline payroll in the selected hubs.

### Sample characteristics

The characteristics of the flight attendants who responded to the survey are shown in Table 1. The mean age of the flight attendants was 47 years; most were female (80%) and 41% had twenty or more years on the job. Over 90% had at least some college education. Only 9% of flight attendants described themselves as current smokers and only 12% reported being overweight. U.S. flight attendant jobs demand moderate flexibility in terms of routes, schedules, and seating capacity and layout according to different types of aircraft. In our cohort, crew seemed to equally share work on both long and short haul flights and multiple aircraft types. While there are differences in occupational exposures according to long haul and short haul flights and type of aircraft, our survey could not separate flight attendants into neat categories. Rather, half of the flight attendants in the survey answered that they worked a combination of long and short segments; 33% stated that they worked single segment, long haul flights; 12% worked multiple segments in one duty period; and flight attendants reported working in multiple types of aircraft over the past 12 months.

**Table 1:** Characteristics of the flight attendant sample

**Characteristic**	**N**	**Percentages with 95% confidence intervals**
Age (Mean = 46.7 ± 9.8 S.D.)	N = 3985	
18 -39		24.6 (23.2-25.9)
40 -59		66.3 (64.8-67.8)
≥ 60		9.1 (8.2-10.0)
Gender	N = 3981	
Male		20 (19-21)
Female		80 (79-81)
Tenure as Flight Attendant	N = 3685	
< 6years		9.8 (8.9-10.8)
6 – 10 years		19.7 (18.4-21.0)
11 - 15 years		12.9 (11.8-14.0)
16 – 20 years		16.1 (14.9-17.2)
>20 years		41.4 (39-8 -43.0)
Education	N = 3977	
<high school diploma		0% (n = 3)
High school or GED		5.4 (4.7-6.1)
Some college, no degree		35.7 (34.2-37.2)
Two-year college degree		14.3 (13.2-15.3)
Four-year college degree		36.6 (35.1-38.1)
Graduate education		7.9 (7.0 -8.7)
Current Smoker	N = 4011	
No		91 (90.1-91.7)
Yes		9 (8.1-9.9)
Overweight/Obese	N = 3877	
No		87.8 (86.7-88.8)
Yes		12.2 (11.2-13.2)

S.D. = Standard Deviation

Comparing the randomly selected sample to the convenience sample showed no noteworthy differences (see Additional file 2: Table S2).

### Health profile of flight attendants

Frequently reported acute and chronic health conditions (reported by at least 15% of all participants) are listed in Table 2. These conditions fall into several major categories: respiratory, neurological, musculoskeletal, auditory, dermatological, and general systems (anxiety/depression, sleep problems, bloating and high blood pressure).

**Table 2:** Prevalence of health conditions reported by at least 15% of flight attendants

	**Percentage of flight attendants (95% confidence intervals)**	**Total number**
***A. FREQUENT SYMPTOMS: lasting 5-7 days (OVER PAST WEEK)***		
Sinus congestion	29.0 (27.6 – 30.5)	3,789
Bloating	25.2 (23.8 - 26.6)	3,750
Fatigue	27.3 (25.9 - 28.7)	3,817
Anxiety	20 (18.7 – 21.3)	3,778
Back pain	27.7 (26.3 – 29.1)	3,787
Foot pain	28.5 (27.1 – 30.0)	3,775
Shoulder/elbow/wrist/hand pain	29.4 (28.0 – 30.9)	3,792
Generalized muscle aches	23.3 (21.9 – 24.7)	3,775
***B. NOTABLE CONDITIONS: needing medical attention (OVER PAST 12 MONTHS)***		
Reactive airways/sinusitis/allergies	54.7 (53.1 - 56.2)	3,850
Shortness of breath/reduced lung capacity	15.5 (14.4 – 16.7)	3,787
Other respiratory symptoms	14.6 (13.4 – 16.7)	3,436
Severe headache	23.4 (22.1 – 24.7)	3,804
Numbness/tingling of extremities	17 (15.8 – 18.2)	3,801
Dizziness/lightheadedness	19.4 (18.1 – 20.6)	3,796
Memory loss/Lack of concentration	14.7 (13.6 - 15.8)	3,783
Fatigue	36.8 (35.3 – 38.3)	3,809
Muscle weakness	16.3 (15.1 – 17.5)	3,778
Joint aches/pains	33.3 (31.8 – 38.8)	3,813
Rashes/hives	15.5 (14.3 – 16.6)	3,805
***C. DIAGNOSED CONDITIONS: told by a care provider (EVER****)*		
High blood pressure	16.7 (15.5 – 17.8)	3,882
Chronic bronchitis	15.6 (14.5 – 16.7)	3,910
Migraines	19.4 (18.2 – 20.6)	3,934
Hearing loss	17 (15.9 - 18.2)	3,853
Low back pain	52.6 (51.0 – 54.2)	3,861
Sleep disturbances	33.7 (32.2 – 35.2)	3,852
Depression/Anxiety	36.3 (34.8 – 37.8)	3,851
Allergies	39.0 (37.5 – 40.6)	3,831

### Health of flight attendants compared to general U.S. Population

Table 3 compares the prevalence of health conditions found in both the flight attendant survey and a survey of the U.S. population (NHANES), adjusted for age and stratified by gender. The NHANES sample excluded unemployed respondents, those below the poverty line, and individuals with less than a high school education in order to reflect the characteristics of the flight attendants.

**Table 3:** Prevalence of health conditions in NHANES survey (2005 -2008) and flight attendants’ health survey (2007)

		**NHANES**				**Flight attendants**				**Standardized prevalence ratio (Age-adjusted)**	
**Reported health conditions**	**Gender**	**% Prevalence**	**Weighted n**	**S.E.**	**95% Confidence interval (CI)**	**% Prevalence**	**n**	**S.E.**	**95% Confidence interval (CI)**	**SPR**	**95% Confidence interval (CI)**
**Respiratory health**											
**Allergies†**	**Male**	31.6	1201	1.7	27.9 – 35.2	35	766	1.7	31.6 – 38.5	1.11	0.98 – 1.25
	**Female**	43.2	1139	1.8	39.3 – 47	40	3035	0.89	38.2 – 41.7	0.89	0.82 – 0.92
**Asthma†**	**Male**	13.2	2432	0.82	11.5 – 14.8	12	781	1.2	9.8 – 14.5	0.94	0.75 – 1.12
	**Female**	15.7	2240	0.92	13.8 – 17.6	13.5	3104	0.6	12.3 – 14.8	0.91	0.82 – 0.99
**Chronic Bronchitis†**	**Male**	3.6	2263	0.5	2.6 – 4.6	13.5	779	1.2	11.2 – 16.1	3.59	2.90 – 4.28
	**Female**	5.1	2083	0.7	3.7– 6.5	16.1	3099	0.7	14.8 – 17.4	2.75	2.51 – 2.99
**Current Smoker**	**Male**	23.6	2262	1.0	21.6 – 25.7	13.2	802	1.2	10.9 – 15.8	0.38	0.31 – 0.45
	**Female**	17.4	2086	1.2	15.0 – 19.7	8.1	3173	0.4	7.1 – 9.1	0.21	0.18 – 0.23
**Cardiac health**											
**Heart Disease†**	**Male**	2.3	2260	0.3	1.6 – 3.0	2.7	768	0.6	1.7 – 4.1	1.39	0.79 – 1.98
	**Female**	0.6	2084	0.2	0.1 – 1.0	2.5	3059	0.2	2.0 – 3.1	3.51	2.72 – 4.30
**High BP†**	**Male**	23.3	2433	1.1	21.1 – 25.6	25	773	1.6	22.0 – 28.2	1.0	0.86 – 1.19
	**Female**	22.3	2238	1.3	19.6 – 25.1	14.6	3077	0.6	13.3 – 15.9	0.54	0.49 – 0.58
**Overweight†**	**Male**	28.3	2432	1.3	25.7 – 30.9	12.6	771	1.2	10.3 – 15.1	0.42	0.34 – 0.51
	**Female**	33.8	2238	1.4	30.9 – 36.7	12.2	3075	0.6	11.1 – 13.4	0.33	0.30 – 0.37
**Mental health**											
**Sleep Disorder†**	**Male**	7.7	2432	0.6	6.5 – 9.0	31.6	766	1.7	28.3 – 35.0	3.69	3.22 – 4.15
	**Female**	5.6	2237	0.6	4.3 – 6.8	34.2	3056	0.9	32.5 – 35.9	5.61	5.27 – 5.95
Fatigue*	**Male**	3	2244	0.4	2.3 – 3.8	6.6	758	0.9	4.9 – 8.6	2.18	1.57 – 2.78
	**Female**	5.9	2065	0.6	4.7 – 7.1	10.6	3028	0.6	9.6 – 11.8	1.83	1.63 – 2.03
Depression*	**Male**	0.6	2243	0.2	0.2 – 1.0	3.7	761	0.7	2.5 – 5.3	5.67	3.57 – 7.77
	**Female**	1.6	2065	0.4	0.9 – 2.4	3.8	2961	0.4	3.2 – 4.6	2.18	1.77 – 2.58
**Other**											
**Reproductive cancer†**											
	**Female**	2.9	2080	0.5	2.0 – 3.9	5.0	3101	0.3	4.3 – 5.8	1.34	1.13 – 1.56

^**†**^Health conditions that were diagnosed by a health care provider

*Symptoms that lasted almost everyday in the past 1-2 weeks as reported by respondent.

Compared to the general U.S. population, flight attendants reported an increased prevalence of chronic bronchitis; males showed a 3.5 fold prevalence [SPR] and females showed 2.75 times the age-adjusted prevalence of chronic bronchitis. This increase in chronic bronchitis was remarkable given the lower prevalence of smoking in flight attendants. In addition, asthma and allergies were significantly less prevalent in female flight attendants. Male flight attendants had similar prevalence rates of asthma and allergies compared to the general population.

Female flight attendants had a 3.5 fold increase in the reported prevalence of cardiac disease compared to the NHANES population even though they had a significantly lower prevalence of hypertension, smoking, and being overweight, known risk factors for heart disease. The prevalence of hypertension and cardiac disease in male flight attendants was similar to the general population despite a significantly lower prevalence of being overweight and smoking.

Male and female flight attendants had 3.7 and 5.7 times the reported prevalence of diagnosed sleep disorders compared to the general population, adjusted for age. In addition, fatigue and depression in female flight attendants were about twice that of the NHANES population.

Male flight attendants also had twice the expected prevalence of fatigue, however, their report of depression that occurred everyday or nearly everyday showed a 5.7 times greater prevalence compared to the general population.

Female reproductive cancers, including breast, uterus, and ovary, were significantly more prevalent in flight attendants compared to the general population; flight attendants showed a thirty-four percent greater prevalence of these cancers.

### Relation between health conditions and Job tenure

Given the increased reported prevalence of some health conditions in flight attendants, we were interested to understand whether the reported prevalence of these conditions changed with longer exposure to the work environment, such as longer job tenure. To test the association between job tenure and the prevalence of disease, we examined only those conditions that were reported as diagnosed by a health provider in order to minimize the bias of subjective report. Table 4 presents the frequently reported diagnoses in flight attendants compared to the general population. For the full results of NHANES from 2005-2006 and 2007-2008, please see the continuous NHANES selected bibliography [37].

**Table 4:** The relationship between job tenure and the prevalence of health conditions in flight attendants adjusted for age, smoking, education, overweight

**Condition**	**Conditional Odds Ratio per 5- year tenure**	**95% CI**	**Standard Error**
**Chronic bronchitis**			
All	1.17	1.07-1.28	.05
Male	1.43	1.14-1.79	.16
Female	1.11	1.01-1.23	.01
**Heart disease**			
All	1.17	.95-1.45	.13
Male	0.95	.63-1.44	.20
Female	1.32	1.01-1.74	.18
**High blood pressure**			
All	1.06	.98-1.16	.04
Male	1.04	.89-1.22	.08
Female	1.13	1.02-1.25	.06
**Sleep disorder**			
All	1.05	.99-1.12	.03
Male	1.13	.97-1.32	.09
Female	1.04	.97-1.12	.04
**Hearing loss**			
All	1.23	1.03-1.22	.05
Male	1.12	1.02-1.23	.05
Female	1.13	.94-1.35	.10
**Reproductive cancer**			
Female	0.91	0.79-1.06	.07
**Skin cancer**			
All	1.30	1.13-1.49	.09
Male	1.35	1.00-1.82	.21
Female	1.27	1.10-1.48	.10
**Migraines**			
All	1.07	.99-1.15	.04
Male	1.06	.84-1.33	.12
Female	1.04	.97-1.12	.04
**Depression/Anxiety**			
All	1.08	1.02-1.16	.03
Male	1.09	.933-1.27	.09
Female	1.07	.999-1.02	.04

Certain pulmonary and cardiac conditions showed an association with job tenure. For example, males had 43% greater odds and females had 17% greater conditional odds of a diagnosis of chronic bronchitis for each five years of tenure, after adjusting for age, smoking, education, and being overweight. Longer tenure increased the risk of heart disease in females by 32% for every five-year increase in tenure, although males had no increased risk. Interestingly, females also had an increased risk of high blood pressure with longer tenure (13% increase for every five years on the job) while males showed no increased risk.

Other notable associations with tenure were skin cancer, hearing loss, and depression/anxiety. Sleep disorders, migraines, and reproductive cancers in females were not associated with tenure.

## Discussion

To our knowledge, this is the largest survey of general health in flight attendants with a comparison to the larger U.S. population [9,21]. We found that, compared to the general population, flight attendants have an increased prevalence of a number of conditions, and some of the leading diagnoses are associated with longer job tenure, even after adjusting for other risk factors, such as age, smoking, education, and Body Mass Index [BMI]. Thus, several findings about flight attendant health warrant attention. Using the NHANES population as a reference, allowed us to 1) compare the health of the general population of the US to domestic flight attendants, 2) control for important SES characteristics (education, poverty, and employment status) and 3) measure similar survey questions without limiting the data to a comparison of one particular occupation.

In a review of studies on flight attendant health, researchers found that most studies are not random samples, were conducted many years ago, rely on self-reported questionnaire data, and suffer from low response rates. While our response rate was 48%, it was higher than we expected and higher than the most recent large, random sample conducted by Ebbert in 2007, which yielded a response rate of 14% [23].

The higher than expected reported prevalence of chronic bronchitis in flight attendants adds further support to studies that have found adverse respiratory outcomes in flight attendants associated with cigarette smoking before it was banned in-flight. The recognition of significant exposure to second-hand tobacco smoke (SHTS) in the cabin led to increasingly stricter smoking bans from 1988 to 1999, at which time, 97% of flights to and from the U.S. were smoke-free [10]. As early as 1989, researchers found elevated levels of urinary cotinine, a tobacco metabolite, evident in crew members during post-flight periods [38]. Models generated from cotinine dosimetry estimated that the flight attendants’ exposure to SHTS was greater than 6 times that of the average worker and approximately 14 times that of the average person [39]. Moreover, at least one study confirmed compromised pulmonary function in 49 flight attendants who never smoked but worked in the aircraft cabin before the ban [40]. Considering that 41% of flight attendants in our study had greater than twenty years on the job, their exposure to SHTS is likely to have been considerable. In addition, the odds of being diagnosed with chronic bronchitis, increased significantly with longer tenure, even after controlling for other risk factors such as age, current smoking, BMI, and education.

Other recent studies of flight attendant health that limited the sample to individuals without a personal history of current or past cigarette smoking found increased prevalence of chronic bronchitis in the crew also. Beatty et al. (2011) compared age-adjusted prevalence of chronic bronchitis in flight attendants to that in the general population in one wave of the NHANES survey and found a prevalence of 11.7 percent in flight attendants versus 7.2 percent in NHANES [21]. In addition, the prevalence of other respiratory illnesses, such as emphysema/COPD and sinus problems was increased in flight attendants. These differences were notable because the NHANES sample in the Beatty et al. study included unemployed individuals with likely a higher prevalence of disease, such that the unemployed sample would include also those too sick to work. Although the researchers found respiratory diseases to be elevated in flight attendants compared to the general population, the prevalence of illnesses did not increase with tenure. This study was limited, however, by a small sample size (n = 235), gross estimates of tenure (tenure in ten-year increments), a relatively older sample, (mean age of 58.2 years), and potentially biased responses (the sample was openly recruited to investigate respiratory health). Nonetheless, the odds of daily respiratory complaints, such as nasal congestion, or throat or eye irritation not related to cold or hay fever, were related to tenure in these never smokers.

A large study (n = 1,007) by Ebbert et al (2007) that selected never smokers randomly found an association between tenure and respiratory illnesses, such as sinusitis, middle ear infection, and asthma [23]. Prevalence of diagnosed chronic bronchitis did not show the same dose-response relationship with tenure, however, despite the high prevalence rate of 30.8% in this population. Importantly, this sample was selected for pre-1987 seniority (older flight attendants exposed to SHTS before the smoking bans) with only partial blinding to the study hypotheses, and the survey had a relatively low response rate of 14%.

Other cabin exposures besides past exposure to SHTS, may contribute to respiratory symptoms in flight attendants. Previous researchers investigated respiratory symptoms associated with ozone toxicity, low humidity and cabin pressure, along with other air contaminants, to explain respiratory symptoms in crew [22,24,27]. Tashkin et al (1983) found increased symptoms of ozone toxicity in crew during flights in aircraft designed to fly at higher altitudes while a later study found no difference in four ozone-related symptoms (coughing, chest tightness, shortness of breath and “breathing hurts”), throughout the flight [25]. Both of these studies investigated symptoms that occurred in-flight only without consideration of possible delayed effects post-flight. Whelan et al. (2003) found flight attendants were more likely than teachers or blue-collar workers in a national survey to report chest illness even though they were less likely than the comparative groups to report a diagnosis of asthma [26]. Importantly, all of these studies were conducted before smoking was completely banned and no direct measurement of exposure was collected.

In the current study, reported cardiac disease prevalence was 3.5 times greater in female flight attendants than the general population. The male flight attendants showed a higher prevalence of cardiac disease as well, although, not significant in this small number of male flight attendants compared to female flight attendants (768 versus 3059). The finding of *any* increase in cardiac disease prevalence was surprising, nonetheless, given the lower reported prevalence in the flight attendants of hypertension (females), smoking, and being overweight. This finding must be considered, also, in light of the slight difference in the cardiac questions between the flight attendant survey and the NHANES survey. The flight attendants were asked if they had been diagnosed with “heart disease” and the NHANES respondents were asked if they had been diagnosed with “coronary artery disease”. These differences may have led to a misclassification of heart disease in both the flight attendants survey and the NHANES survey. In a sensitivity analysis, we found evidence of some confusion about cardiac diagnoses in that only 50% of respondents who reported a diagnosis of myocardial infarction (heart attack) also reported a diagnosis of heart disease (flight attendants) or coronary artery disease (NHANES). In other words, myocardial infarction was not interpreted as either “heart disease” or “coronary artery disease” half of the time, showing some confusion about cardiac diagnoses. Notably, the prevalence of myocardial infarction in both groups was very rare in this selection of employed populations or “healthy workers”. Despite these limitations, heart disease in female flight attendants showed an exposure-response relationship with tenure, as did hypertension, a major risk factor for heart disease, even after adjustment for smoking, age, being overweight, and education.

Several other exposures in the cabin environmental have been associated with cardiac disease, including air pollution, noise, and sleep disruption. First, exposure to SHTS and ozone, risk factors for respiratory disease, have been shown also to increase the risk of cardiac disease.^34^ Second, recent evidence from population studies indicates that chronic exposure to occupational noise may increase the risk for cardiac disease [41]. Airplane noise has been measured at an average of 80 to 85 decibels [42], with higher sound pressure levels during engine start and takeoff, and some researchers have noted an increased risk of hearing loss in cabin crew with exposures between 71 and 81 decibels [43]. In our study, the reported diagnosis of hearing loss in flight attendants showed a exposure-response relationship with tenure after controlling for age and other factors. Third, circadian disruption that results from shift work and crossing time zones has been demonstrated in flight attendants using melatonin as a biomarker [44] and, based on new research, chronic circadian disruption may increase the risk for cardiac disease [45,46]. In the current study, flight attendants reported significantly higher prevalence of diagnosed sleep disorders than the general public, even though the exposure-response relationship with tenure was not significant. Underscoring a problem with sleep, 37% of the flight attendants surveyed reported having sought medical attention for frequent fatigue within the past year. Overall, cardiac disease in flight attendants could be increased by a number of factors including air contaminants in the cabin, noise and circadian disruption.

Although other studies have reported problems with fatigue and depression in flight attendants, this is the first study to compare these reports to results from a national sample, such as NHANES. The higher than expected prevalence of fatigue and depression in flight attendants was surprising, given that only flight attendants reporting fatigue and depression everyday in the last week were compared with a decidedly more liberal definition in NHANES; individuals experiencing these symptoms “nearly every day” in the past 2 weeks. The different time interval and frequency criteria, such as, “daily” symptoms in last week (flight attendants) versus symptoms that occurred “nearly every day” over past 2 weeks (NHANES) may be a conservative estimate of the flight attendant experience in comparison. Furthermore, a diagnosis of depression in flight attendants showed a moderate exposure-response relation with job tenure.

Previous research about cancer diagnoses in flight attendants is complicated by equivocal findings. In our study, we found an increased reported prevalence of reproductive cancers, inclusive of breast, ovary, and uterus, in female flight attendants. In addition, the report of a diagnosis of skin cancer in flight attendants was significantly associated with tenure in the job. These study results contrast with a recent study of cancer in 11,311 former flight attendants which found no evidence for an increased occurrence of breast cancer or melanoma, although, this study investigated only mortality rates in a cohort considerably different from our study sample [15]. In particular, the median tenure of flight attendants was only 5.9 years compared to our study in which 41.4% of the flight attendants had more than twenty years in the job. Nonetheless, Paridou et al. found also no elevated risk of cancer mortality in a Greek cohort of 843 pilots and 1,835 cabin crew [14]. A recent cohort study of flight attendant health did not find breast cancer incidence (morbidity versus mortality) significantly different compared to NHANES, however, flight attendants in this study were not selected randomly and were not compared with employed persons only. The NHANES sample included unemployed persons too, a group less likely to be healthy [21]. Other cohort studies of female flight attendants did find higher than expected incidence of both breast cancer and melanoma [17] in California, Iceland and Sweden, although the elevated risk of breast cancer in Swedish crew was not significant and was not associated with length of employment [13,16]. Further, two separate meta-analyses of published incidence studies also found elevated risk for breast cancer and melanoma [11,18].

In considering the results of our study in total, it is important to recognize that a cross-sectional survey study is not meant to explain cause and effect. Yet, the higher than expected age-adjusted prevalence of health conditions in flight attendants would suggest that occupational exposures may contribute to the problems. Our results describe a U.S. flight attendant population and may not reflect the same experience for crew of foreign airline carriers. The SPR is an indirect standardization measure, and like the SMR, should not be compared across studies unless stratum specific ratios are nearly constant across strata or when stratum specific population sizes are the same for the study and reference population. An additional limitation to this work is that both the FA survey and selected NHANES questions rely on self-reported health conditions; these data were not corroborated with medical records due to the cost and scope of the work. Furthermore, another limitation to this work is the use of tenure as a proxy for exposure; similar to most occupational settings, the flight attendants in this study likely do not have uniform exposures over time and between participants.

## Conclusions

This study has identified several significant health conditions in flight attendants compared to the general population and raises the important issue about what can be done to minimize risk. While smoking bans have limited some occupational exposures, many questions about hazardous exposures still exist. Importantly, flight attendants do not have access to exposure data such as cabin air quality, or noise and radiation levels. Compared with most of the U.S. workforce, flight attendants are not covered by the Occupational Safety and Health Administration (OSHA) regulations. Instead, the Federal Aviation Administration (FAA) oversees health and safety protections. While both agencies have had a memorandum of understanding to work together to achieve equal worker protections for several decades, only recently (August 27, 2013) did FAA issue a final policy [47] to give OSHA partial jurisdiction over flight attendant safety and health on aircraft, starting with the application of OSHA’s rules for hearing protection, hazard communication, and blood-borne pathogens. The FAA also formally acknowledged that OSHA’s injury/illness recordkeeping, employee access to exposure records, and whistleblower protections already apply to flight attendants.

Sleep disorders in flight attendants significantly affected nearly one in three flight attendants in our study. This finding is important because of the consequences for health (particularly the risk for cardiovascular disease), quality of life, productivity, and public safety. Not surprisingly, Congress called for the Civil Aeronautical Medical Institute (CAMI) within FAA to study the problem in 2005 and 2008. CAMI researchers found that disrupted sleep activity between off-duty and on-duty work cycles resulted in pervasive chronic sleep deprivation, fatigue, and decline in tests of cognitive performance among flight attendants [7]. CAMI cited the key variables with the potential to reduce risk of fatigue as the total length of duty day, number of flight legs/segments per day, recovery time in the hotel during a trip, consecutive duty days/trip length, and number of days off in between trips. Although not mentioned by CAMI, work factors such as the physical stress of hypobaric hypoxia at altitude [48], workload, and noise may fatigue also [49]. Currently, FAA considers limits on duty time for fatigue mitigation [50] choosing a focus on work/rest cycles instead of the best practices based on sleep/wake factors [8]. In all, the management of fatigue and sleep disruption still needs to be fully addressed by the airlines or the FAA.

Musculoskeletal pain is widely reported in our sample. Frequent musculoskeletal pain reported by one-third of the flight attendants matches the results of other studies [51]. Importantly, no studies have tracked musculoskeletal complaints over time as passenger loads have climbed along with population obesity, full occupancy policies, smaller passenger seating, and new baggage charges that influence the type and number of passenger carry-on bags. These changes may challenge crew who work in these restricted cabin quarters and may increase their physical demands as they attempt to squeeze people and belongings into tight spaces. We know little about the consequences of these ergonomic conditions, especially aboard new “Very Large Transport Aircraft” such as the Airbus 380 or the Boeing 787-10. Future studies are needed in this area.

Finally, the prevalence of general neurological symptoms in an otherwise healthy worker population is curious and potentially concerning. Reports of severe headaches, dizziness or lightheadedness, numbness and tingling in extremities, and memory loss, are difficult to gauge because we did not have comparable survey questions in the NHANES survey or other worker surveys. Potential exposures that have been associated with neurological effects are important starting points for future investigation and include exposure to neurotoxic oil-based chemicals in the cabin air supply, hypoxia, and overexposure to pesticide products applied either during or prior to some international flights.

In summary, the prevalence of certain health conditions in flight attendants is higher than the general population and some of these conditions show an exposure-response relationship with tenure. While FAA assumed the responsibility for the occupational health and safety of cabin crew starting in 1975, the agency has published few regulations for flight attendants since then, such that the scope of health protection programs for flight attendants is limited in comparison to other worker groups covered under OSHA.

## Abbreviations

NHANES: National Health and Nutrition Examination Surveys; NCHS: National Center for Health Statistics; CDC: Centers for Disease Control and Prevention; SPR: Standardized prevalence ratio; SHTS: Secondhand tobacco smoke; BMI: Body mass index; CAMI: Civil Aeronautical Medical Institute; FAA: Federal Aviation Administration; OSHA: Occupational Safety and Health Administration.

## Competing interests

This study was funded in part by the Federal Aviation Administration, Research grant No. 2005-G-014 Although the FAA has sponsored this project, it neither endorses nor rejects the findings of this research.

## Authors’ contributions

EM, IT, LK, JB, and SH designed and conducted the flight attendant health survey. EM, SG, IT, BC conducted the NHANES analysis. EM and SG drafted the manuscript and all authors contributed in its content.

## Supplementary Material

Additional file 1: Table S1 FA Survey 2007 variables compared to NHANES 2005-2008 questionnaire variables. Click here for file

Additional file 2: Table S2 Distribution of random sample versus convenience sample. Click here for file
