# Supplementary material for: The self-reported health of U.S. flight attendants compared to the general population
Source: Environ Health. 2014 Mar 10;13:13. doi: 10.1186/1476-069X-13-13 (PMC4007523; doi:10.1186/1476-069X-13-13)
Supplement: Additional file 1: Table S1 — FA Survey 2007 variables compared to NHANES 2005-2008 questionnaire variables. [file 1476-069X-13-13-S1.doc]

Table S1: FA Survey 2007 variables compared to NHANES 2005-2008 questionnaire variables

| Variable | FA Survey 2007 | | NHANES 2005-2006 | | NHANES 2007-2008 | |
| --- | --- | --- | --- | --- | --- | --- |
|  | Name | Description | Name | Description | Name | Description |
| Allergy | H15au | Ever told by health care provider allergies? | AGD040 | Doctor or other health professional ever told you that you have allergies | - | - |
| Asthma | H15l | Ever told by health care provider have asthma? | MCQ010 | Doctor or other health professional ever told you that you have asthma | MCQ010 | Doctor or other health professional ever told you that you have asthma |
| Chronic Bronchitis | H15k | Ever told by health care provider chronic bronchitis? | MCQ160K | Doctor or other health care professional ever told you that you had chronic bronchitis | MCQ160K | Doctor or other health care professional ever told you that you had chronic bronchitis |
| Smoker | H18 | Ever smoked more than 100 cigarettes in your lifetime? | SMQ020 | Smoked at least 100 cigarettes in life | SMQ020 | Smoked at least 100 cigarettes in life |
| Current Smoker | H18a | Currently smoke or smoke in past? | SMQ040 | Do you now smoke cigarettes? | SMQ040 | Do you now smoke cigarettes? |
| Coronary heart disease | H15c | Ever told by health care provider have heart disease? | MCQ160C | Doctor or other health care professional ever told you that you had coronary heart disease | MCQ160C | Doctor or other health care professional ever told you that you had coronary heart disease |
| High Blood Pressure | H15b | Ever told you had high blood pressure | BPQ020 | Have you ever been told by a doctor or other health professional that you had hypertension, also called high blood pressure | BPQ020 | Have you ever been told by a doctor or other health professional that you had hypertension, also called high blood pressure |
| Overweight | H15a | Ever told by health care provider--overweight/obesity? | MCQ080 | Doctor or other health professional ever told you that you were overweight | MCQ080 | Doctor or other health professional ever told you that you were overweight |
| Sleep Disorder | H15ao | Ever told by health care provider have sleep disorder? | SLQ060 | Ever told by doctor or other health professional that you have a sleep disorder? | SLQ060 | Ever told by doctor or other health professional that you have a sleep disorder? |
| Fatigue | H13z | Past week, unusual tiredness or fatigue | DPQ040 | Over the last 2 weeks--Feeling tired or having little energy | DPQ040 | Over the last 2 weeks--Feeling tired or having little energy |
| Feeling depressed | H13ab | Past week depressed mood | DPQ020 | Over the last 2 weeks--Feeling down, depressed, or hopeless | DPQ020 | Over the last 2 weeks--Feeling down, depressed, or hopeless |
| Cancer: |  |  | MCQ220 | Have you ever been told by a doctor or other health professional that you had cancer or malignancy of any kind | MCQ220 | Have you ever been told by a doctor or other health professional that you had cancer or malignancy of any kind |
| Reproductive: Breast/Ovary/Uterus | H15r | Ever told by health care provider reproductive cancer (i.e. breast, ovary,uterus)? | MCQ230A-D | 14,28,38 What Kind—Breast, Ovary, Uterus | MCQ230A-D | 14,28,38 What Kind—Breast, Ovary, Uterus |
| **Demographics:** |  |  |  |  |  |  |
| Age | H16 | How old are you? | RIDAGEYR | Age at Screening Adjudicated - Recode | RIDAGEYR | Age at Screening Adjudicated - Recode |
| Gender | H17 | What is your gender? | RIAGENDR | Gender | RIAGENDR | Gender |
| Education | H20 | How much schooling have you completed? | DMDEDUC2 | Education Level - Adults 20+ | DMDEDUC2 | Education Level - Adults 20+ |
| Poverty | - | - | INDFMPIR | Ratio of family income to poverty threshold | INDFMPIR | Ratio of family income to poverty threshold |
| Employment | - | - | OCD150 | Which of the following were you doing last week (1) working at a job or business, (2) with a job or business but not at work, (3) looking for work, or (4) not working at a job or business?” | OCD150 | Which of the following were you doing last week (1) working at a job or business, (2) with a job or business but not at work, (3) looking for work, or (4) not working at a job or business?” |
