# Supplementary material for: The self-reported health of U.S. flight attendants compared to the general population
Source: Environ Health. 2014 Mar 10;13:13. doi: 10.1186/1476-069X-13-13 (PMC4007523; doi:10.1186/1476-069X-13-13)
Supplement: Additional file 2: Table S2 — Distribution of random sample versus convenience sample. [file 1476-069X-13-13-S2.doc]

Table S2: Distribution of random sample versus convenience sample

| **Sample**  **Characteristic** | | **Percent of Convenience Sample**  **(n=1,398)** | **Percent of**  **Random Sample**  **(n= 2,613)** |
| --- | --- | --- | --- |
| Age | 18-39 years | 27 | 23 |
|  | 40-59 years | 65 | 67 |
|  | ≥ 60 year | 8 | 9 |
| Gender | Male | 23 | 18 |
|  | Female | 77 | 81 |
| Tenure | <6 years | 9.8 | 9.7 |
|  | 6-10 years | 20.6 | 19.2 |
|  | 11-15 years | 14.6 | 12.1 |
|  | 16-20 years | 14.1 | 17.1 |
|  | ≥20 years | 41 | 42 |
